# Efficacy of Topiramate Alone and Topiramate Plus Vitamin D3 in the Prophylaxis of Pediatric Migraine: A Randomized Clinical Trial

**Published:** 2020

**Authors:** Razieh FALLAH, Saeedreza SARRAF YAZD, Seid Mojtaba SOHREVARDI

**Affiliations:** 1 Department of Pediatrics, Growth Disorders of Children Research Center, Shahid Sadoughi University of Medical Sciences, Yazd, Iran; 2 Department of Clinical Pharmacology, Faculty of Pharmacy, Shahid Sadoughi University of Medical Sciences, Yazd, Iran

**Keywords:** Migraine, Child, Prophylaxis, Vitamin D_3_, Topiramate

## Abstract

**Objective:**

Topiramate is effective in the prevention of pediatric migraine, and studies show that vitamin D_3_ supplementation might also be useful in the treatment of adult migraineurs with a normal vitamin D_3_ level. The present study aimed at comparing the efficacy and safety of topiramate plus vitamin D_3_ and topiramate alone in the prophylaxis of pediatric migraine.

**Materials & Methods:**

In a single-blinded, randomized, clinical trial, 5-15-year-old children with migraine headaches, referred to the Pediatric Neurology Clinic of Shahid Sadoughi Medical Sciences University, Yazd, Iran from January 2016 to January 2017, were randomly allocated to receive 2 mg/kg/day of topiramate or 2 mg/kg/day of topiramate plus one 500,000 IU vitamin D_3 _pearl weekly for two consecutive months.

Primary outcomes were the reduction of monthly frequency, severity, duration, and the disability score of migraine, and the secondary outcomes included a good response to treatment (more than 50% reduction in monthly headache frequency) and a lack of clinical adverse events.

**Results:**

Totally, 31 female and 26 male children with the mean age of 10.02±2.11 years were evaluated. Both drugs were effective in the reduction of monthly frequency, severity, duration, and disability for headaches. Nevertheless, the combination of topiramate and vitamin D_3_ was more effective than topiramate alone in reducing the monthly headaches frequency (6.12±1.26 vs. 9.87±2.44 times, P=0.01) and disability score (19.24±6.32 vs. 22.11±7.91, P=0.02). Good response to treatment was observed in 60.7% and 75.9% of the subjects in the topiramate alone and topiramate plus vitamin D_3_ groups, respectively, and topiramate plus vitaminD3 was more effective (P= 0.01). Transient mild side effects were observed in 14.3% and 17.2% of the subjects in the topiramate alone and topiramate plus vitamin D_3_ groups, respectively (P=0.8).

**Conclusion:**

A combination of topiramate and vitamin D_3_ might be considered safe and more effective than topiramate alone in the prophylaxis of pediatric migraine.

## Introduction

Migraine, as the most common primary headache in children, occurs in up to 10.6% of the 5-15-year-old children and migraine prophylaxis should be started if headache episodes occur more than once a week or the disability score is higher than 20 based on the pediatric migraine disability assessment scale (pedMIDAS)(1). There is no unique guideline for the prophylaxis of pediatric migraine, although the US Food and Drug Administration has approved flunarizine and, recently, topiramate for migraine prophylaxis in pediatric patients (2), and topiramate, as a safe and effective drug, should be strongly encouraged for migraine prevention in children (3). The efficacy of topiramate in migraine prophylaxis is proved in Iranian children (4-6).

Today, physicians are interested in using alternative medicine and non-pharmacological remedies for migraine prevention, and many nutraceutics, such as magnesium, coenzyme Q10, riboflavin, butterbur, feverfew, melatonin, etc., are utilized for the prophylaxis of migraine in children and adults (7). 

Vitamin D_3_ deficiency (serum 25-hydroxyvitamin D_3_ or 25-OH vitamin D_3_ level <20 ng/mL) and insufficiency (25-OH vitamin D_3_ <30 ng/mL) are worldwide public health issues (8) that can influence all body organs with different complications, including myopathies, cancers, skin, musculoskeletal, autoimmune, cardiovascular, and kidney diseases, as well as mental disorders, such as depression and schizophrenia (9,10). In a study in Tehran, Iran, 86% of 9-12-year-old children had vitamin D_3_ deficiency (11). 

Association between the dysfunction of transporter proteins of vitamin D_3_ metabolites and migraine attacks was observed in a study by Nagata et al. (12), and that higher levels of vitamin D_3_ might reduce the risk of migraine headaches. In some researches, patients with non-migraine headaches had low serum levels of 25-hydroxyvitamin D_3_ (10, 13, 14). Association between migraine and serum 25-hydroxyvitamin D_3_ level was not observed in some studies (13, 15, 16). However, in a study in Isfahan, Iran, a positive relationship was observed between serum levels of vitamin D_3_ and migraine (17), and in another study in Tehran, adult migraineurs had a lower serum vitamin D_3_ level and vitamin D_3_ deficiency, and insufficiency was more frequent in them compared to healthy controls. It was concluded that the increase in serum levels of 25-hydroxyvitamin D_3_ might decrease the risk of migraine headaches (18). In a study in Turkey, serum vitamin D_3_ level was lower in patients with migraine (19). Association between the prevalence of migraine and the latitude was observed, and the higher latitude had a higher one-year prevalence for migraine. Moreover, the presence of vitamin D receptor, 1alpha-hydroxylase, and vitamin D-binding protein in the hypothalamus was suggestive of the role of vitamin D_3_ deficiency in the incidence of migraine headaches (20).

A randomized clinical trial by Gazerani et al., showed the prophylactic efficacy of 100 μg/day supplement of vitamin D_3_ for the prevention of migraine in 18-65-year-old migraineurs, and the drug was more effective than placebo in decreasing daily headache episodes and frequency, and 25-OH vitamin D_3_ levels significantly increased in the vitamin D_3_ group during the first 12 weeks of treatment (21).

The efficacy of vitamin D_3_ therapy in adult migraineurs (22, 23), as well as 8-16-year-old children with migraine headaches (24), was reported in a few research and there is limited efficacy evidence of vitamin D_3_ therapy in the prophylaxis of pediatric migraine. Further randomized clinical trials are required to evaluate this issue.

The present study aimed at answering the question that whether or not topiramate plus vitamin D_3_ is more efficient than topiramate alone in reducing the monthly frequency, severity, and the disability score in 5-15-year-old migraineur children without vitamin D_3_ deficiency.

## Materials & Methods

In a randomized, single-blind, clinical, open-label, parallel-group study, the efficacy of topiramate alone and topiramate plus vitamin D_3_ was compared in the prophylaxis of migraine headaches of 5-15-year-old children without vitamin D_3_ deficiency, referred to the Pediatric Neurology Clinic of Shahid Sadoughi Medical Sciences University, Yazd, Iran from January 2016 to January 2017. All participants were the Yazd City inhabitants and equally exposed to direct sunlight.

A more than 50% decrease in the monthly headache frequency during the follow-up period was defined as a good response to treatment. The sample size was determined 30 in each group using the Z formula, based on the confidence interval (CI) of 95%, power of 80%, type I error of 5%, and the effect size (difference in the frequency of good response between the two groups) of 30% for the primary endpoint. 

Inclusion criteria were: children aged 5-15 years with migraine headaches based on the International Headache Society Criteria (25), one or more headache attacks per week, moderate or severe disability for headaches or the PedMIDAS score >20 (26), normal hemoglobin (Hb) level and mean corpuscular volume (Hb level >11.5 g/dL, mean corpuscular volume >80 fL), sufficient vitamin D_3_ serum level (>30 ng/mL), not taking vitamin D_3_ supplements within the past two months, and not receiving any migraine preventive therapies.

Exclusion criteria were: the presence of systemic diseases (renal, cardiac, hepatic, hemostatic, diabetes mellitus, etc.) based on clinical and laboratory screening evaluations, headaches other than migraine or secondary headaches, excessive drugs usage, discontinuation of drugs for more than one week, allergy to topiramate or vitamin D_3_, and vitamin D_3_ serum level of >80 ng/mL (27).

The trial used computer-generated random numbers for the simple randomization of subjects, and the allocation ratio was 1:1 for the two groups. Randomization and blinding were performed by an investigator not clinically involved in the trial. Data collectors, outcome assessors, data analysts, and the staff delivering the intervention were blind to the allocation. The drug was delivered by the nurse of the clinic and was packed and labeled according to a medication code schedule generated before the trial. Inside all packages, the amount of the drug in one tablet and its dosage were written. After opening the packages, the drug dosage was determined by the pediatric neurologist, based on the weight of the child. Primary and secondary outcomes were assessed by the pharmacy student of the research not aware of the drug group assignment (6).

Totally, 60 children were randomly assigned to two groups to receive 2 mg/kg/day of topiramate or 2 mg/kg/day of topiramate plus one 500,000 IU vitamin D_3 _pearl weekly for two consecutive months. In the current study, 500,000 IU vitamin D_3 _pearls and 25 and 50 mg topiramate tablets were purchased from Daroupakhsh Co., Iran.

The patients were visited weekly for two consecutive months by the pharmacy student of the research in the Pediatric Neurology Clinic of Shahid Sadoughi Hospital and clinical information about frequency, severity, and duration of headaches, the pedMIDAS score (26), and frequency and severity of clinical side effects of the drugs were recorded by interviewing the mothers and reviewing self-reported diaries and daily notes of parents. The severity of headaches was assessed by asking each child to grade the majority of headache pain on the 11-point visual analogue scale (VAS) (28) from 0 as no pain to 10 as the most severe pain. The VAS is a 10-cm long horizontal or vertical line, marked at the ends with no pain and worst pain imaginable, and the child is asked to place a mark on the line that represents his pain level. 

Safety evaluation included spontaneous reports of side effects, physical exam, and assessment of vital signs. Parents were requested to call the researcher promptly if serious adverse events (i.e., refractory nausea or vomiting, topiramate-related heatstroke, drug rashes with eosinophilia and systemic symptoms, severe anorexia and weight loss, difficulties with concentration, behavior changes, abdominal pain or renal colic, lethargy, and disorientation) occurred.

Throughout the research, analgesics (acetaminophen or ibuprofen) were permitted for the symptomatic relief of moderate to severe headache attacks.

The drugs were continued for 60 consecutive days and, then, monthly frequency, severity, and duration of headaches, and the pedMIDAS scores before and after three months of intervention were compared. A more than 50% reduction in monthly headaches frequency was considered as a good response to treatment (27).

Primary outcomes were the frequency of good response to treatment (more than 50% reduction in monthly headaches frequency), and reduction in severity, duration, and disability for headaches. The secondary outcome was the clinical side effects of drugs.

The data were analyzed in SPSS version 17. The Chi-squared or Fisher exact test was used for the data analysis of qualitative variables, and mean values were compared by t-test. Differences were considered significant at P-values <0.05.

Informed consent was taken from parents before the administration of drugs, and the study protocol was approved by the Ethics Committee of Shahid Sadoughi University of Medical Sciences. The study was not funded by any pharmaceutical companies. The current study was registered in the Iranian Registry of Clinical Trials (registration number: IRCT201701092639N20).

## Results

Two children in the topiramate alone and one in the topiramate plus vitamin D_3_ groups discontinued drug usage during the follow-up period, and finally, the trial was completed with 57 children, 31 females (45.6%) and 26 males (54.4%), with the mean age of 10.02 ± 2.11 years.

The comparison of some characteristics of the children in both groups is shown in Table 1, indicating that age and gender distribution, as well as the type and positive family history of migraine, were not significantly different between the two groups.

Table 2 shows the comparison of headaches characteristics before treatment between the two groups, indicating that the monthly frequency, severity, duration, and disability for headaches were not significantly different between the groups.

Comparisons of headaches characteristics before and after the treatment in the topiramate alone and topiramate plus vitamin D_3_ groups are presented in tables 3 and 4, showing that both drugs were effective in the reduction of monthly frequency, severity, duration, and disability for headaches.

Table 5 shows the comparison of headaches characteristics after treatment, indicating that topiramate plus vitamin D_3_ was more effective than topiramate alone in the reduction of monthly frequency and disability score. The severity and duration of headaches were not significantly different between the two groups.

After eight weeks of treatment, good response (more than 50% reduction in the frequency of the monthly headaches) was observed in 17 children in the topiramate alone (60.7%; 95%CI: 42.93%-79.07%) and 22 subjects in the topiramate plus vitamin D_3_ (75.9%; 95%CI: 60.46%- 91.54%) groups, and topiramate plus vitamin D_3_ was significantly more effective (P=0.01).

 Clinical side effects were observed in 14.3% (n=4) of the topiramate alone group subjects, including daily sleepiness in two, anorexia in one, and difficulty with concentration in one child, and in 17.2% (n=5) of the patients in the topiramate plus vitamin D_3_ group, including daily sleepiness in two, constipation in two, and anorexia in one child. The safety of both drugs had no significant difference (P =0.8). All the adverse effects were transient and disappeared in one or two weeks, and treatment was stopped in none of the patients who developed side-effects. 

**Table. 1:** Comparison of the Characteristics of Children in the Study Groups

Group		Topiramate Alone	Topiramate Plus Vitamin D_3_	P-value
Data				
Age, yr (mean ± SD)		10.67 ± 2.11	10.33 ± 2.45	0.79
Gender	Female	16	15	0.7
	Male	12	14	
Type of migraine	Without aura	20	19	0.9
	With aura	8	10	
Positive family history of migraine	Yes	22	24	0.8
No	6	5

**Table. 2:** Comparison of Headache characteristics Before Treatment in the Study Groups

Group	Topiramate Alone	Topiramate Plus Vitamin D_3_	P-value
Data			
Monthly headaches frequency (mean ±SD)	12.98±3.98	13.43±4.11	0.5
The severity of headaches (mean ±SD)	6.97±2.11	6.82 ± 2.9	0.9
Headaches duration, h (mean ±SD)	2.31 ± 0.52	2.22 ± 0.63	0.6
Disability for headaches: pedMIDAS score (mean ±SD)	32.12 ± 8.89	32.23 ± 9.45	0.7

**Table. 3:** Comparison of Headaches Characteristics Before and After Treatment in the topiramate Alone Group

Group	Before treatment (Mean ±SD)	After treatment (Mean ±SD)	P-value
Data			
Monthly headaches frequency	12.98±3.98	9.87 ± 2.44	0.01
The severity of headaches	6.97±2.11	4.89 ± 2.01	0.04
Headaches duration, h	2.31 ± 0.52	1.39 ± 0.11	0.01
Disability for headaches: pedMIDAS score	32.12 ± 8.89	22.11 ± 7.91	0.001

**Table. 4:** Comparison of Headaches Characteristics Before and After Treatment in the Topiramate Plus Vitamin D_3_ Group

Group	Before Treatment (Mean ±SD)	After Treatment (Mean ±SD)	P-value
Data			
Monthly headaches frequency	13.43±4.11	6.12±1.26	0.001
The severity of headaches	6.82 ± 2.9	4.72 ± 1.99	0.01
Headaches duration, h	2.22 ± 0.63	1.44 ± 0.86	0.0001
Disability for headaches: pedMIDAS score	32.23 ± 9.45	19.24 ± 6.32	0.0001

**Table .5:** Comparison of Headaches Characteristics After Treatment in the Study Groups

Group	Topiramate (Mean ±SD)	Topiramate Plus Vitamin D_3_ (Mean ±SD)	P-value
Data			
Monthly headaches frequency	9.87 ± 2.44	6.12±1.26	0.01
The severity of headaches	4.89 ± 2.01	4.72 ± 1.99	0.5
Headaches duration, h	1.39 ± 0.11	1.44 ± 0.86	0.1
Disability for headaches: pedMIDAS score	22.11 ± 7.91	19.24 ± 6.32	0.02

## Discussion

 Many drugs are used for the prophylaxis of pediatric migraine. In the study by Wheeler et al., 40.7% of the patients with chronic migraine had vitamin D_3_ deficiency (29). Exposure of arms and legs or arms and face two or three times per week to direct sunlight can be associated with a sufficient vitamin D_3_ level. Nevertheless, many factors, including age, obesity, melanin level, application of sunscreens, wearing covered dresses, drugs, time of the day, latitude, and sun exposure through glass can change the production of vitamin D in the skin. Hyperthyroidism and hypothyroidism can also severely affect the vitamin D_3_ level (30). Vitamin D_3_ may have a role in the secretion of serotonin and dopamine, while the two neurotransmitters are involved in migraine pathogenesis. Vitamin D receptors present in the hypothalamus, as a region for migraine pain sensation, and the neuroprotective and antioxidant effects of vitamin D_3_ in the central nervous system can support the relationship between vitamin D_3_ and prophylaxis of migraine (24, 31). Association between low serum levels of vitamin D_3_ and more incidence of chronic headaches, and the effectiveness of vitamin D_3_ in the treatment of a few headache disorders are reported (10, 20, 32, 33). 

Some studies reported that the incidence of migraine attacks increases during autumn and winter in children, or a seasonal change in vitamin D_3_ level is correlated with an increase in migraine attacks, which mostly occurs in winter (20, 24, 34). 

In the present study, the efficacy and safety of topiramate plus vitamin D_3_ and topiramate alone in the prophylaxis of pediatric migraine were compared. Effectiveness and tolerability of low-dose topiramate in the prevention of migraine in children were shown in a study by Fallah et al. (5). 

Based on the results of the present study, the combination of 2 mg/kg topiramate and 500,000 IU vitamin D_3 _weekly for two consecutive months was more effective than topiramate alone in reducing the monthly frequency and disability score of migraine in 5-15-year-old children without vitamin D_3_ insufficiency (serum 25-OH vitamin D_3_ >30 ng/mL). Also, in a Turkish study, the combination of 1 mg/kg amitriptyline and 400 IU per day vitamin D_3_ for six months was more effective than amitriptyline alone in headaches frequency of 6-18-year-old children with migraine and normal serum 25-OH vitamin D_3_ level (>20 ng/mL) (24).

The limitations of the present study were lack of placebo, short duration of treatment, and lack of follow-up after discontinuation. 

## Conclusions

Results of the current study showed that the combination of topiramate and vitamin D_3_ was more effective than topiramate alone in the reduction of monthly frequency and the disability score of migraine in children, and vitamin D_3_ therapy might be considered a safe and effective strategy for the prophylaxis of pediatric migraine. Further clinical trials with larger sample sizes, altitude control, and seasonal differences are required to determine the optimal dose of vitamin D_3_ for the prevention of pediatric migraine. 
